# Current insights into the molecular mechanisms of hypoxic pre- and postconditioning using hypobaric hypoxia

**DOI:** 10.3389/fnins.2015.00388

**Published:** 2015-10-23

**Authors:** Elena Rybnikova, Mikhail Samoilov

**Affiliations:** Laboratory of Neuroendocrinology, and Laboratory of Regulation of Brain Neuron Functions, Pavlov Institute of Physiology, Russian Academy of SciencesSt. Petersburg, Russia

**Keywords:** hypoxic preconditioning, hypoxic postconditioning, neuroprotection, hypoxic tolerance of the brain, molecular mechanisms

## Abstract

Exposure of organisms to repetitive mild hypoxia results in development of brain hypoxic/ischemic tolerance and cross-tolerance to injurious factors of a psycho-emotional nature. Such preconditioning by mild hypobaric hypoxia functions as a “warning” signal which prepares an organism, and in particular the brain, to subsequent more harmful conditions. The endogenous defense processes which are mobilized by hypoxic preconditioning and result in development of brain tolerance are based on evolutionarily acquired gene-determined mechanisms of adaptation and neuroprotection. They involve an activation of intracellular cascades including kinases, transcription factors and changes in expression of multiple regulatory proteins in susceptible areas of the brain. On the other hand they lead to multilevel modifications of the hypothalamic-pituitary-adrenal endocrine axis regulating various functions in the organism. All these components are engaged sequentially in the initiation, induction and expression of hypoxia-induced tolerance. A special role belongs to the epigenetic regulation of gene expression, in particular of histone acetylation leading to changes in chromatin structure which ensure access of pro-adaptive transcription factors activated by preconditioning to the promoters of target genes. Mechanisms of another, relatively novel, neuroprotective phenomenon termed hypoxic postconditioning (an application of mild hypoxic episodes after severe insults) are still largely unknown but according to recent data they involve apoptosis-related proteins, hypoxia-inducible factor and neurotrophins. The fundamental data accumulated to date and discussed in this review open new avenues for elaboration of the effective therapeutic applications of hypoxic pre- and postconditioning.

Identification of the molecular and physiological mechanisms underlying the response of organisms to environmental factors, in particular, to harmful injurious exposures represents one of the major research problems in biology and medicine. To solve this problem is fundamentally important for further elucidation of the endogenous mechanisms responsible for adaptation to the environment, as well as for elaboration of the effective tools to increase the resistance of the organism and its most vulnerable organs, the heart and brain, to detrimental external and internal factors.

The central place in the formation of various types of adaptive reactions of the organism to environmental factors belongs to the neural process of perception and transduction of the adaptogenic signal which the famous Russian physiologist and Nobel prize laureate Ivan Pavlov designated as a “warning” signal. One of Pavlov's disciples P. K. Anokhin further developed the concept of “alarm” or “warning” activity as the basis for the *phenomenon of anticipatory reflection of reality* which is a universal phenomenon of life allowing to “anticipate the course of future events in order to better adapt to the environment” (Anokhin, 1974). According to modern concepts, in the nervous system the process of adaptation to possible damaging effects can manifest itself by the following cascade: an adaptogenic stimulus (“warning” signal, specifically injurious or stress factors of mild intensity) induces reorganization of the plasticity of the elements of the nervous system (neurons, synapses, glia). At the root of this reorganization lies the processing of anticipatory reflection of reality which prepares brain cells to the expected deleterious exposures and is associated with an induction of evolutionarily-acquired gene-determined protective mechanisms. As a result, reprogramming of death/survival mechanisms in the brain cells is activated leading to neuroprotection and compensation of the deleterious effects. By means of such cascade events, the “warning” signal results in environmentally-determined adaptive change in the phenotype, and the process represents an illustrative example of hormesis or neurohormesis. Hormesis is a basic principle of physiology which is generally defined as responses of cells or organisms to a factor which induces stimulatory or beneficial effects at low doses and inhibitory or adverse effects at high doses (Calabrese et al., 2010; Calabrese and Mattson, 2011). A preconditioning phenomenon aimed at adaptation to extreme factors is one of the typical examples of the adaptogenic “warning” signaling and, therefore, of hormesis. Thus, it is likely that various types of the preconditioning, as well as other hormetic stimuli, share some common protective mechanisms. From this point of view, elucidation of the mechanisms launched by the hypoxic preconditioning can be important in a broader context, shedding more light on the basic phenomenon of hormesis.

## The phenomenon of preconditioning

The terms “preconditioning” and “tolerance” were introduced into experimental practice in 1964 (Janoff, 1964). At the end of the last century Murry and colleagues demonstrated that multiple brief ischemic episodes might actually protect the myocardium from a subsequent sustained ischemic insult, and this phenomenon has been termed “ischemic preconditioning” (Murry et al., 1986). In the 1990s a hypoxic/ischemic tolerance induced by pre-exposure to brief (sublethal) *preconditioning* ischemic episodes has been described and partially studied in the brain (Kitagawa et al., 1990; Kirino et al., 1991). In the next 25 years the mechanisms of cerebral ischemic preconditioning have been uncovered in various *in vivo* and *in vitro* models including global and focal brain ischemia, surviving brain slices, cultured primary neurons and transient ischemic attacks in humans (for review see Kirino, 2002; Steiger and Hänggi, 2007; Obrenovitch, 2008; Shpargel et al., 2008; Gidday et al., 2013). According to the current concept, formation of brain ischemic tolerance induced by ischemic preconditioning consists of two sequential phases (“windows”): the early (an induction of tolerance) and the late (an expression of tolerance) phase (Kirino, 2002; Steiger and Hänggi, 2007; Stenzel-Poore et al., 2007; Shpargel et al., 2008). The first window involves rapidly-induced (from a few minutes to several hours) changes in the cells such as activation of protein kinases, proteases, posttranslational modifications of ion channel proteins, receptors, redox-sensitive proteins and, apparently, immediate early gene transient expression. In the second window (24 h and later) the delayed mechanisms associated with gene expression and *de novo* synthesis of proteins providing long-lasting plasticity and neuronal survival are being activated.

## Hypoxic preconditioning

Compared to ischemic preconditioning, effects and mechanisms of preconditioning by mild or moderate hypoxic or anoxic episodes (hypoxic preconditioning) have been understudied although this paradigm obviously represents a convenient method of treatment since it does not require surgery. The phenomenon was first described in the 1960s as “a kind of induced tolerance of tissue-cells to hypoxia” (Lu, 1963). The neuroprotective action of brief anoxic pre-exposure was reported in 1964 (Dahl and Balfour, 1964) almost 30 years earlier than cerebral ischemic precondition had been described. Further progress in developing the concept of hypoxic (anoxic) preconditioning and brain tolerance has been achieved in the *in vitro* electrophysiological studies on rat hippocampal slices performed by Schurr and colleagues (Schurr et al., 1986).

One of the earliest models for studying hypoxic preconditioning was autohypoxia. This represents a whole-body preconditioning when anesthetized rodents are placed into an individual sealed container, and authohypoxia is induced by the animal's own oxygen consumption (Lu, 1963). When such a procedure is repeated up to 5 times, the animal becomes more tolerant because the survival time (as judged by the onset of gasping) was found to increase up to 8 times as compared to control non-preconditioned animals (Lu et al., 1999).

Following this paradigm, the most common technique for studying hypoxic preconditioning was the use of 8–13% normobaric hypoxia. To achieve this, animals are usually placed in a hypoxic chamber in which oxygen content is decreased by substituting it with nitrogen. Humans can inhale such a hypoxic gas mixture through a mask (Serebrovskaya et al., 2011), which represents a non-invasive way of application and a more beneficial technique compared to the ischemic methods with regard to their implementation in clinical practice. Although the therapeutic window of the normobaric hypoxia preconditioning appears to be rather narrow lasting only approximately 72 h (Stetler et al., 2014), it has been reported that various regimes of normobaric hypoxic preconditioning significantly protect animals against injurious effects of subsequent global or focal stroke (Miller et al., 2001), kainic acid-induced seizures and brain edema (Emerson et al., 1999a,b). Numerous developmental studies applying normobaric hypoxic preconditioning at the early stages of ontogenesis demonstrated neuroprotection from ischemic brain injury in different peri- and postnatal periods (Gidday et al., 1994; Vannucci et al., 1998).

*In vitro* models of cerebral hypoxic preconditioning include studies in hippocampal slices (Pérez-Pinzón et al., 1996; Bickler and Fahlman, 2009) and olfactory cortex (Semenov et al., 2002; Samoilov et al., 2003), as well as primary neuronal cultures (Arthur et al., 2004). It has been demonstrated that preconditioning with a single short (2 min) episode of anoxia in slices of the olfactory cortex or three trials of 1 min anoxia in the hippocampal slicesincreases the resistance of the cells to severe “test” anoxia, preventing depression of the evoked potential amplitudes and calcium overload associated with anoxic injury whereas moderate calcium load is required for induction of tolerance during anoxic preconditioning (Pérez-Pinzón et al., 1999; Semenov et al., 2012).

## Hypobaric hypoxic preconditioning

Another promising technique of hypoxic preconditioning with high translational potential is exposure to mild hypobaric hypoxia. The revitalizing effects of moderate high altitude occurring in nature have been known for centuries. Currently it is well-established that adaptation (acclimatization) to high altitude both of natural origin and simulated in the hypobaric chamber has a number of beneficial effects on human health, whole body hypoxic tolerance and improvement of physical training (Meerson, 1984; Meerson et al., 1996; Millet et al., 2013). These effects are observed under conditions of chronic mild hypobaric hypoxia (acclimatization mode) and result from reprogramming of the cardio-pulmonary and metabolic processes, including erythropoiesis, vascular remodeling, pulmonary changes and cardiac hypertrophy (Hultgren and Miller, 1967; Savourey et al., 2004; Brito et al., 2015). The integrity of these reactions is called systemic “structural trace of adaptation” due to which the resistance of an organism against any forthcoming hypoxic exposures is achieved along with more general protective effects (Meerson, 1984).

Hypobaric hypoxia is also widely used in the mode of intermittent (multiple, interlaced by normoxic periods) hypoxic training adapted as a therapeutic strategy to treat heart diseases, allergy (Meerson et al., 1987) and for training of athletes to improve their sea-level performance (Levine, 2002). In experimental practice the neuroprotective and anticonvulsant potential of chronic intermittent hypobaric hypoxia has also been demonstrated (Gong et al., 2012; Zhen et al., 2014).

In our laboratory during several years we have been using an original technique of hypobaric hypoxic preconditioning which was developed in early 2000s and consistently validated in subsequent studies (Samoilov et al., 2001; Rybnikova et al., 2005, 2006). With this method, three trials of mild hypobaric hypoxia (360 mm Hg, equivalent to 10% of normobaric oxygen and an altitude of 5 km above sea level, 2 h duration of each episode) spaced at 24 h are necessary and sufficient to achieve the most pronounced neuroprotective effect and significantly protect the brain against subsequent severe global hypoxia. Such a mode of hypoxic preconditioning has greatly improved survival of rats during severe hypoxia (3 h, 180 mm Hg, equivalent to 5% of normobaric oxygen and an altitude of 11 km) and prevented hypoxia-induced injury/loss of vulnerable brain neurons in the hippocampus and neocortex, as well as diminished functional disturbances observed at the behavioral level (Samoilov et al., 2001; Rybnikova et al., 2005). Surprisingly, preconditioning by three episodes of mild hypobaric hypoxia has also demonstrated a potent antidepressant-like and anxiolytic action and protected animals from development of stress-related depressions and anxiety (Rybnikova et al., 2007a). The neuroprotective efficacy of such a preconditioning mode against an ischemic brain injury was further proved by another research group in a model of global cerebral ischemia in gerbils (Duszczyk et al., 2009) and also in a model of cardiac arrest and resuscitation (Xu and Lamanna, 2014).

## Cerebral mechanisms of hypoxic preconditioning

A central place in the studies of hypoxic preconditioning belongs to identification of its molecular mechanisms with special attention being paid both to their unique character and similarity with the mechanisms of ischemic preconditioning. The process of preconditioning can be divided into several phases starting with the phase of initiation of hypoxic tolerance (Samoilov, 1997; Samoilov et al., 2003) or the immediate phase of adaptation to hypoxia (Lukyanova et al., 2013) which covers the first few minutes following the exposure to moderate hypoxia. This acute stage is clearly linked with the launch of several molecular signaling processes. The important mechanism which significantly contributes to the development of the later phases of brain hypoxic tolerance is obviously associated with the acute changes in neuronal redox state in favor of reduction equivalents that, in turn, can result in modifications of calcium bound to intracellular hydrophobic components (for review see Samoilov et al., 2003). Another key process at this stage is a remodeling of the respiratory mitochondrial chain and switching to the mitochondrial complex II, as well as rapid and transient activation of hypoxia-inducible factor HIF-1 (Lukyanova et al., 2009, 2012). Succinate-induced stabilization of HIF-1α followed by an activation of the transcriptional factor HIF-1 and expression of its target pro-adaptive genes is currently considered as one of the main mechanisms contributing to development of the long-term adaptation to hypoxia (Lukyanova et al., 2009).

The second phase of hypoxic preconditioning, the phase of induction of long-term hypoxic tolerance, involves significant alterations of the intracellular signal transduction processes, primarily modest activation of the glutamatergic, calcium, phosphoinositide, cyclic AMP regulatory systems (Semenov et al., 2000, 2002; Samoilov et al., 2003) and rapid changes in pro- and antioxidant reactions (Ravati et al., 2000: Furuichi et al., 2005). This is followed by fast protein kinase- and protease-dependent modifications of ion channels, receptors, redox-sensitive proteins, as well as triggering of the third phase—the phase of expression of hypoxic tolerance. The question arises what signal mechanisms are responsible for the transformation of the hypoxic tolerance induction phase into the phase of its long-term expression.

As in cerebral ischemic tolerance, formation of the delayed phase of brain tolerance (*thephase of expression*) induced by hypoxic preconditioning is associated with activation of pro-adaptive genes and expression of their products which affect intracellular plasticity readjustment aimed at retention of vital neuronal functions and structural integrity. A principal role in controlling genome activity belongs to so-called third messengers—transcription factors which are activated in the cytosol, translocated to the nucleus and which bind to the promoters of target genes regulating their transcription (Morgan and Curran, 1991). The transcription factors are activated by intracellular components of calcium, phosphoinositide, cyclic AMP and pro-oxidant regulatory systems and therefore link two sequential phases of hypoxic/ischemic tolerance development, namely the phases of its induction and expression.

The main activators of the late-response genes engaged in the mechanisms of neuronal plasticity and survival include inducible (c-Fos, NGFI-A, HIF-1) and ubiquitous (pCREB, NF-κB) transcription factors. Inducible transcription factors are products of immediate early genes whose expression is driven by signaling cascade-dependent remodeling of chromatin enzymes and modifications of histones, as well as by interaction with ubiquitous transcription factors (Morgan and Curran, 1991; Sng et al., 2004). The hypoxia-inducible factor HIF-1α is stabilized and activated under hypoxic conditions due to hypoxia-dependent inactivation of prolyl hydroxylase reactions and is also controlled by NF-κB, NGFI-A, MAP/ERK (Kallio et al., 1999; Samoilov et al., 2007). Ubiquitous transcription factors CREB, NF-κB are activated by phosphorylation and factors of cellular stress (cytokines, reactive oxygen species, Ca^2+^_i_) (Mattson et al., 2000; Ravati et al., 2000; Kitagawa, 2007).

**Figure 1:**
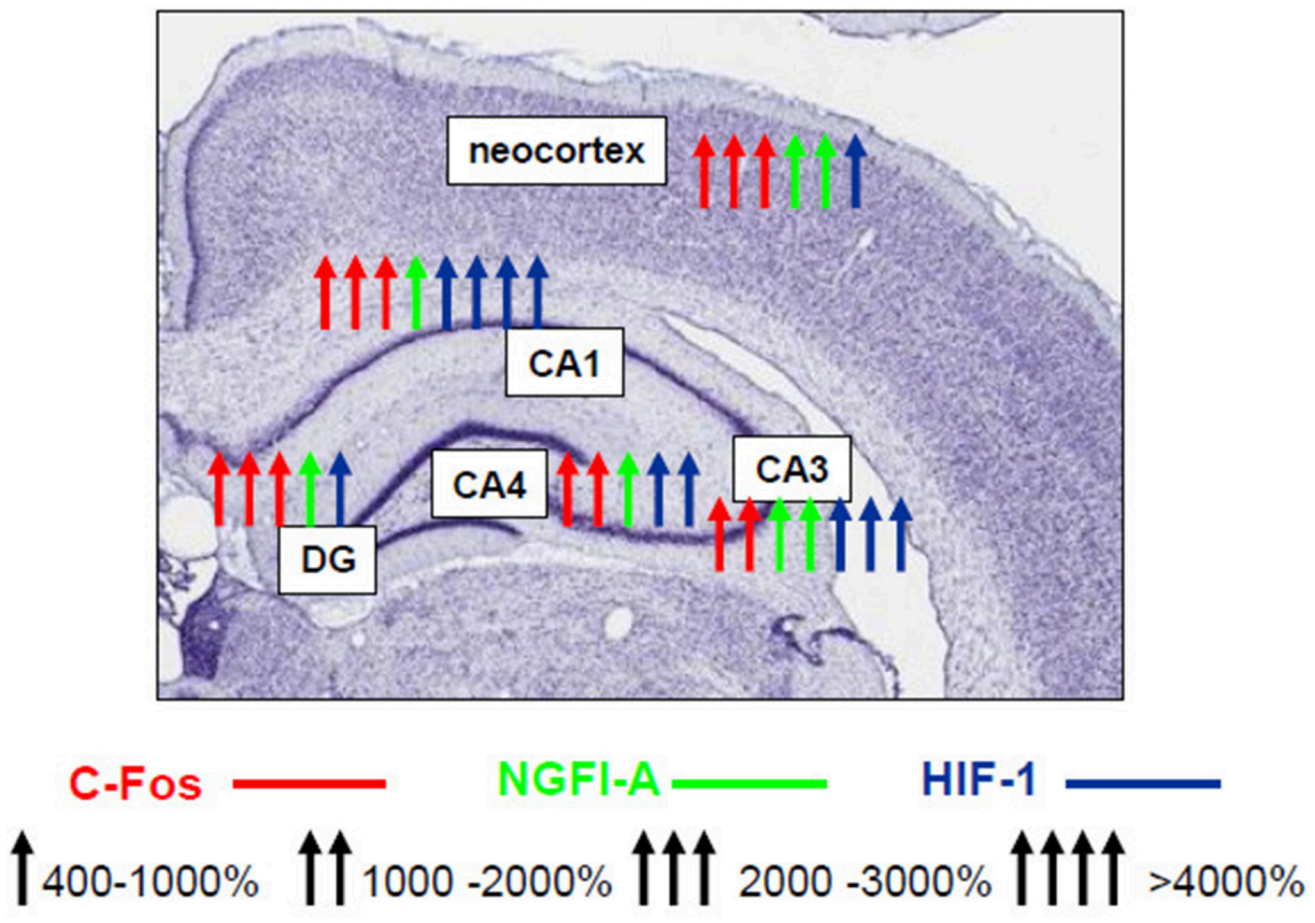
**Schematic representation of activating effect of hypobaric hypoxic preconditioning on the transcription factors NGFI-A, c-Fos, HIF-1 in various areas of hippocampus and neocortex (Rybnikova et al., 2009, 2015)**.

Until recently, only scattered non-systematic data on the nature of the involvement of various transcription factors in the mechanisms of expression of brain hypoxic tolerance have been reported. Using our model of hypobaric hypoxic preconditioning, a complex study of the expression and activity profiles of the key transcription factors in the rat brain has been performed over the years which allowed us to conclude that neuroprotective hypoxic preconditioning by three episodes of mild hypobaric hypoxia induces persistent (up to 24 h) neuronal overexpression of inducible (c-Fos, NGFI-A, HIF-1α) and activation of ubiquitous (pCREB, NF-kB) transcriptional factors in the most vulnerable brain regions including the fronto-parietal neocortex and various hippocampal fields (CA1, CA3/4, dentate gyrus) (Rybnikova et al., 2008a, 2009). Although we have observed some specific changes characteristic to the neocortex and different areas of the hippocampus (Figure 1) it is noteworthy that despite the different levels of expression of the transcription factors their cooperative “cross” activation was observed in all examined brain structures in preconditioned rats.

More recent results indicate that the cooperative induction of transcription factors can be attributed to the hypoxia-tolerant phenotype depending on the efficacy and mode of hypoxic preconditioning. Strong activation of neuronal CREB (its phosphorylation) and NF-κB p65 in the neocortex has been observed following the three-trial but not the single-trial preconditioning (Samoilov et al., 2014). In contrast to the three-trial preconditioning, the single preconditioning trial does not result in formation of the hypoxia-tolerant phenotype and provides no protection against the structural and functional injury produced by severe hypoxia.

It is well-known that downstream targets for the transcription factors NGFI-A, c-Fos, HIF-1α, pCREB, and NF-κB include genes encoding pro-adaptive proteins: neurotrophins, antioxidants, anti-apoptotic factors of the *bcl-2* superfamily, heat shock proteins, erythropoietin and others, involved in the processes of neuroplasticity and neuronal survival during harmful exposures (Sheng and Greenberg, 1990; Finkbeiner, 2000; Mattson et al., 2000; Semenza, 2000; Rice et al., 2002; Merelli et al., 2013, etc.). Based on this knowledge it can be hypothesized that the cooperative activation of these transcription factors in the brain (“warning” signaling at the level of the genome) promote the most intense expression of the pro-adaptive genes which are essential for preventing the injury of the susceptible brain neurons caused by damaging factors.

One of the most important common mechanisms of ischemic and hypoxic preconditioning involves reprogramming of apoptosis-related processes. Both types of preconditioning up-regulate expression of anti-apoptotic proteins of the Bcl-2 superfamily and repress the levels of Bax (Rybnikova et al., 2006; Liu et al., 2009). A complex of survival-enhancing, neuroprotective and neuroplasticity processes is induced by hypoxic preconditioning in the phase of expression at the level of late-response genes and *de novo* synthesis of the protein products. The most substantial contributions to the hypoxia-tolerant phenotype have been ascribed to the up-regulation of mitochondrial and cytosolic antioxidant enzymes (Lin et al., 2003; Arthur et al., 2004; Stroev et al., 2004, 2005; Shao and Lu, 2012), neurotrophin BDNF (Samoilov et al., 2014), erythropoietin (Bernaudin et al., 2000, 2002a; Grimm et al., 2006), adrenomedullin (Tixier et al., 2008), the dampening of the excitotoxicity by down-regulation of AMPA receptors (Chang et al., 2006) and stimulation of group I metabotropic glutamate receptors (Semenov et al., 2012). A target of HIF-1 vascular endothelial growth factor VEGF was also shown to be intensely up-regulated (Bernaudin et al., 2002b) which appears to promote extensive neurovascular remodeling as described for hypoxic preconditioning recently (Boroujerdi and Milner, 2015). Among other protective mechanisms, an induction of heat shock proteins, in particular Hsp70 has also been demonstrated in a model of hypobaric hypoxic preconditioning (Wang et al., 2012). Adenosine content and adenosine A1 receptor affinity in the hippocampus of rats preconditioned by repetitive autohypoxia are markedly higher than those in non-preconditioned animals or animals preconditioned by a single trial (Zhang and Lu, 1997). Comparing the changes in the neuropeptide Y levels in gerbil hippocampus in two models of preconditioning, hypobaric hypoxic and ischemic, Duszczyk and colleagues have demonstrated that ischemic preconditioning caused a 20% rise in the expression of neuropeptide Y which lasted for 4 days, whereas hypobaric hypoxic preconditioning according to our protocol resulted in a two-fold increase in the neuropeptide Y levels that was maintained for at least 7 days (Duszczyk et al., 2009).

Our recent study has also reported that hypoxic preconditioning differentially alters the expression of specific proteins called ADAMs (a disintegrin and metalloprotease). ADAMsare a family of membrane-anchored glycoproteins capable of shedding a multitude of proteins from the cell surface (van Goor et al., 2009). Various ADAMs act as crucial modulators of physiological and pathophysiological processes and are supposed to contribute to control of neuronal death/survival. Hypoxic preconditioning induces prominent overexpression of ADAM17 mRNA and protein in the hippocampus and neocortex and prevented severe hypoxia-induced up-regulation of ADAM 15 linked to the neuronal injury (Rybnikova et al., 2012a). These findings might be of special importance in the focus of neurodegeneration since some of the ADAMs including ADAM10 and ADAM17 (TACE) function as amyloid precursor protein (APP) α-secretases (Allinson et al., 2003). For this reason, the observed up-regulation of ADAM17 corresponded to a higher level of the soluble form of APP produced in response to hypobaric hypoxic preconditioning may predict some therapeutic properties for this technique in prevention of Alzheimer's disease. The restoration of the ischemia-reduced α-secretase activity and expression of the amyloid-degrading enzymes neprilysin and endothelin-converting enzyme has been reported in other models of hypoxic preconditioning (Nalivaeva et al., 2004).

A specific group of ligand-operated transcription factors is represented by receptors of corticosteroid hormones. There are two subtypes of such receptors differing in their characteristics, affinity, localization and functions, namely glucocorticoid and mineralocorticoid receptors. Both receptor subtypes are found in the brain and play important roles in adaptive behavior and endocrine regulation, in particular, in feedback control of the hypothalamic-pituitary-adrenal axis (HPA) exerted by glucocorticoid hormones. To date the functions of gluco- and mineralocorticoid receptors, and especially of their balance, have been expanded to participation in the processes of neuronal death/survival and neuroplasticity (de Kloet et al., 1998; De Kloet et al., 2005; Almeida et al., 2000; Rogalska, 2010). Our studies have demonstrated that three trials of hypoxic preconditioning considerably affected expression of gluco- and mineralocorticoid receptors in the dorsal and ventral hippocampus, achieving their optimal balance for neuronal survival (Rybnikova et al., 2011) while the single-trial preconditioning exhibited such an effect to a much lesser extent.

The preconditioning-induced changes in brain corticosteroid receptors appear to contribute to the improvement of adaptive capabilities of the whole organism. This is supported by the analysis of survival rates in preconditioned vs. non-preconditioned rats under the conditions of acute lethal hypoxia. As mentioned above, hypoxic preconditioning in our model of hypobaric hypoxia significantly decreased the lethality of rats during 3 h of exposure to severe hypoxia (down to 15%). This allowed us to suggest that such preconditioning results in mobilization of systemic mechanisms of adaptation, primarily of those associated to the functioning of HPA responsible for organization of the adaptive stress response (Selye, 1950). Further evidence substantiating this suggestion has been obtained in our experiments showing that effective hypoxic preconditioning enhances the activity and reactivity of HPA to mild stresses and potentiates the negative glucocorticoid feedback (Rybnikova et al., 2007b, 2008b, 2011). No such effect has been seen for the non-effective preconditioning mode. Very recently Feng and Bhatt (2015) have also described similar changes in HPA activity in the model of normobaric hypoxic preconditioning and shown that the glucocorticoid receptor blocker RU486 significantly inhibited hypoxic PC induced neuroprotection in newborn rats (Feng and Bhatt, 2015). Hence it is obvious that one of the most important and universal protective mechanisms launched by the hypoxic preconditioning is the modification of HPA functioning aimed at the optimal mobilization of the pro-adaptive defenses. It is interesting to note that modifications of HPA appear to represent a specific target for the adaptogenic effects of high altitudes, since they are observed also during high altitude acclimatization and training (Meerson et al., 1996).

In addition to normobaric and hypobaric hypoxic preconditioning, molecular mechanisms of neuroprotection have been extensively studied in the autohypoxic model. In contrast to other models of hypoxic or ischemic preconditioning, a number of pro-adaptive factors were down-regulated in the brains of rats preconditioned by episodes of autohypoxia, including pERK1/2, calcium, excitatory amino acids, nitric oxide, expression of alpha synuclein (Shao and Lu, 2012). On the other hand, enzyme antioxidants, CREB-dependent signaling, and expression of HIF-1 and its target molecule VEGF are up-regulated in the vulnerable brain regions as has been reported for other models. These findings suggest that autohypoxic preconditioning is a model which shares with other models some common mechanisms but differs in its specific features.

An important role of systemic mechanisms for whole-body models of hypoxic preconditioning (autohypoxia, hypobaric hypoxia) might be expected due to an apparent contribution of remote conditioning. The remote preconditioning is a phenomenon in which brief hypoxic/ischemia and reperfusion of one organ increases tolerance in a remote organ (for review see Przyklenk and Whittaker, 2013). In addition, the remote ischemic preconditioning increased resistance of the whole organism to hypobaric hypoxia at high altitude, preventing development of high altitude diseases (Berger et al., 2015). Until recently this phenomenon has been attributed only to the ischemic models (Vijayakumar et al., 2015), however accumulating evidence suggests that in addition to transient ischemia other triggers of remote protection should be introduced, including peripheral nociception, direct peripheral nerve stimulation and electroacupuncture (Heusch et al., 2015). Taking in consideration that during hypoxic preconditioning episodes in the whole-body models all organs and tissues of the body are exposed to the preconditioning hypoxia, systemic mechanisms of remote preconditioning are likely to contribute to the increased tolerance of the brain. A review of the literature gives evidence for both humoral and neural mechanisms of remote preconditioning (Meller and Simon, 2015). Plausible candidates of humoral mediators include plasma microRNA-144 and ATP-sensitive potassium channels of red blood cells (Gopalakrishnan and Saurabh, 2014; Li et al., 2014) but their importance for protecting the brain has not been demonstrated. In contrast to cardioprotection, for neuroprotection resulting from the remote preconditioning there is more evidence on the neural mechanisms, because blocking neural inputs to the central nervous system prevented remote-preconditioning-induced brain protection (Malhotra et al., 2011; Meller and Simon, 2015). Surprisingly, remote ischemic preconditioning, in contrast to our model of hypobaric hypoxia and normobaric hypoxic preconditioning in the newborn, failed to induce a detectable increase in circulating cortisol as recently reported by Birkelund and colleagues (Birkelund et al., 2015).

New and perspective avenues in research into hypoxic preconditioning and brain tolerance involve epigenetic regulation. Recently, it has been reported that methylation of DNA may be involved in the neuroprotection achieved by hypoxic preconditioning (Zhang et al., 2014). The role of other epigenetic mechanisms in the hypoxic preconditioning has not been studied although an involvement of changes at the epigenetic level in cellular responses to hypoxia/ischemia, in particular in the brain, became a subject of several studies (Watson et al., 2010; Perez-Perri et al., 2011; Melvin and Rocha, 2012; Schweizer et al., 2013; Wu et al., 2013; Tsai and Wu, 2014). As noted above, a key role in the formation of preconditioning-induced brain tolerance belongs to the cooperative activation of several transcription factors, HIF-1α being one of the most important. It is known that HIF-1 requires co-activators which modify chromatin structure, opening the access to DNA for the regulators of transcription. HIF-dependent co-activators include histone acetyltransferases, CREB-binding protein (CBP) and p300 (Ema et al., 1999), some histone deacetylases (Kato et al., 2004; Ellis et al., 2009; Seo et al., 2009) and a chromatin remodeling complex SWI/SNF (Wang et al., 2004). Binding of CBP to the CRE elements of gene promoters dependent on pCREB activity is also up-regulated by hypoxic preconditioning, as described above. Thus, involvement of epigenetic mechanisms can be currently expected both at the level of protein-protein interactions with transcriptional regulators and the acetylation of histones followed by chromatin relaxation. However, this hypothesis requires further experimental substantiation, which we are currently working on.

## Hypoxic postconditioning

Intensive research into ischemic preconditioning in the 1990s–2000s inspired establishment of a cognate protective phenomenon—ischemic postconditioning. It has been demonstrated that repetitive brief ischemia applied *after* prolonged coronary occlusion, during early reperfusion (such a mode has been termed *postconditioning)* is cardioprotective by attenuating reperfusion after myocardial injury (Zhao et al., 2003). A link to the brain was suggested later, in 2007 (Zhao, 2007), and the first experimental studies of this research group described the neuroprotective effects of ischemic postconditioning in stroke with a putative role suggested for Akt signaling (Gao et al., 2008) that is similar to the mechanisms of ischemic preconditioning. Further studies on cerebral ischemic postconditioning to date provide controversial results and many issues remain to be solved regarding its possible protective effects and feasibility (Leger et al., 2015).

A neuroprotective potential of hypoxic postconditioning has been thoroughly described for the first time using chronic intermittent normobaric hypoxia in mice (Leconte et al., 2009). It has been demonstrated that later application of hypoxia (1 h, 8% O_2_, 1–5 days) after the focal ischemia significantly reduced delayed thalamic atrophy. The same study reported that hypoxia *in vitro* (0.1, 1, or 2% O_2_) performed 14 h after oxygen glucose deprivation induced neuroprotection in primary neuronal cultures and pointed out the causal mechanistic role for HIF-1α and its target genes erythropoietin and adrenomedullin in this process.

Recently, based on our original studies of hypobaric hypoxic preconditioning, we developed a model of hypobaric hypoxic postconditioning in rats which involves mild hypoxic exposures (360 mm Hg, 2 h) spaced at 24 h applied either 3 or 24 h after severe hypoxia. It was found that both early and delayed applications of the postconditioning episodes remarkably improved recovery from severe hypoxia and attenuated posthypoxic neuronal injury, reducing pyknosis, hyperchromatosis, and interstitial brain edema, as well as the rates of neuronal loss in the hippocampus and neocortex. Besides that, the delayed postconditioning exerted a potent anxiolytic effect on rat behavior, preventing development of posthypoxic anxiety. Both modes of postconditioning had a beneficial effect on the functioning of HPA, but only the delayed postconditioning completely returned HPA to its baseline activity and reactivity to stress (Rybnikova et al., 2012b). Furthermore, such a mode of postconditioning enhanced expression of HIF-1α and erythropoietin in the hippocampal CA1 neurons of rats surviving after severe hypoxia (Vetrovoy et al., 2014). The modifications of apoptosis-related processes and up-regulation of the neurotrophins (BDNF) has also been implicated in the compensatory mechanisms induced by hypobaric hypoxic postconditioning (Vetrovoy et al., 2015). In parallel to our studies, it has recently been reported that hypobaric hypoxic postconditioning reduces brain damage and improves antioxidative defense in the rat model of birth asphyxia (Gamdzyk et al., 2014).

## Conclusions

**Figure 2:**
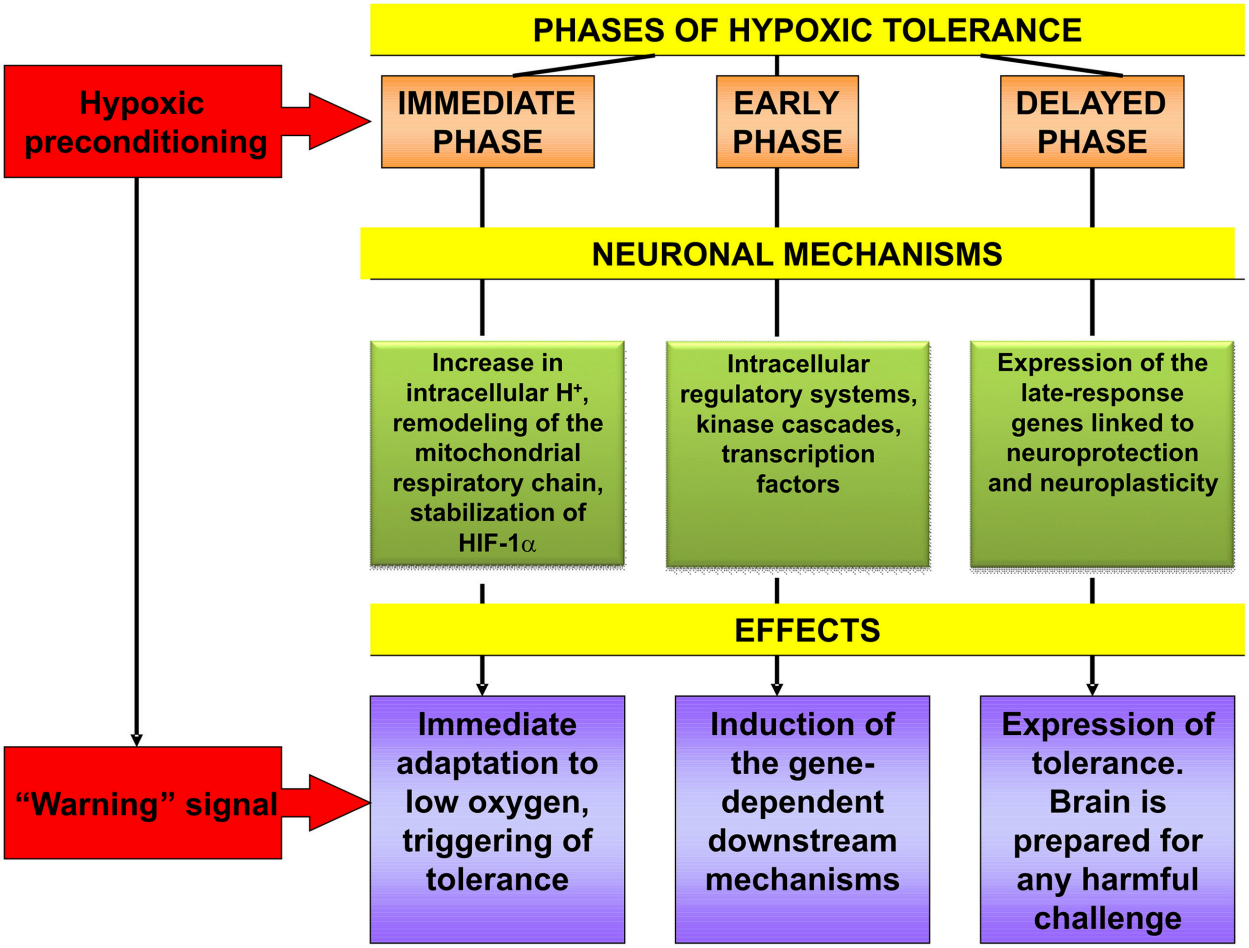
**Cascade of molecular mechanisms involved in brain hypoxic tolerance induced by hypoxic preconditioning**. See references in the text relevant to this Figure.

To summarize the facts and hypothesis discussed above, it should be stated that hypoxic preconditioning functions as a “warning” signalization which mobilizes evolutionarily acquired genome-determined urgent defense mechanisms of brain neurons and the organism as a whole. The processes resulting in their increased resistance to injurious challenge involve induction of multiple intracellular signal components, as well as adjustment of the neuroendocrine control of HPA function. Hypoxic preconditioning-induced cascade mechanisms of intracellular signaling include changes at the level of intracellular redox status, mitochondrial respiratory chain, key second messenger systems, receptor machinery, transcription factors, early and late-response genes which are sequentially engaged in the initiation, induction and expression of the brain hypoxic tolerance (Figure 2). A cooperative activation of the ubiquitous, ligand-operated and inducible transcriptional factors plays the key role for development of the rigorous phase of tolerance expression. Studying the impact of epigenetic regulation in mediating the neuroprotective effects of hypoxic pre- and postconditioning represents a novel perspective trend in this research. Although it is apparent that the main neuroprotective mechanisms of hypoxic pre- and postconditioning might be overlapping this hypothesis needs further investigation.

Based on the notable recent progress in our current knowledge of hypoxic pre- and postconditioning, it is obvious that these techniques possess high translational potential and can inspire development of novel feasible neuroprotective tools for prophylaxis and treatment of neurological, neurodegenerative and stress-related disorders, as well as for increasing of the adaptive capacities of the organism and the brain. It is necessary to consider that adjustment of the particular mode of hypoxic pre- and postconditioing elaborated on the basis of fundamental mechanistic data is principally important for the effective application of their protective potential.

## Author contributions

ER wrote the review. MS developed the whole conception of the review and substantially contributed to the editing of the final version.

### Conflict of interest statement

The authors declare that the research was conducted in the absence of any commercial or financial relationships that could be construed as a potential conflict of interest.
